# Protocol for a multicenter, randomized, single-blind, non-inferiority trial comparing nalbuphine hydrochloride versus hydromorphone hydrochloride for analgesia in mechanically ventilated ICU patients

**DOI:** 10.3389/fphar.2026.1795531

**Published:** 2026-04-28

**Authors:** Yujing Zhang, Yin Yuan, Lehao Ren, Wen Jiang, Shengwen Sun, Rui Wang, Zhuanyun Li, Qiwei Peng, Rui Gong, Xiaobo Yang, You Shang

**Affiliations:** Department of Critical Care Medicine, Union Hospital, Tongji Medical College, Huazhong University of Science and Technology, Wuhan, China

**Keywords:** analgesia, hydromorphone hydrochloride, intensive care, mechanical ventilation, nalbuphine hydrochloride

## Abstract

**Introduction:**

Despite the established use of nalbuphine hydrochloride in anesthesia and obstetrics, high-quality evidence supporting its application in the intensive care unit (ICU) remains limited. This study aims to assess the efficacy and safety of nalbuphine hydrochloride for analgesia in ICU patients.

**Methods:**

This is a multicenter, randomized, single-blind, parallel, positive control study. Eligible patients are randomly assigned to receive nalbuphine hydrochloride or hydromorphone hydrochloride in a 1:1 ratio. Nalbuphine hydrochloride is compared against hydromorphone hydrochloride which serves as the positive control. The study drug infusion is discontinued after 24 h (±10 min) of administration.

**Planned Outcomes:**

The primary outcome is the analgesic success rate during 24 h of study drug administration. The secondary outcomes include the following: 1) The percentage of time within CPOT score ≤2 and RASS score between −2 and +1 during 24 h of study drug administration. 2) The frequency and dosage of rescue sedation during 24 h of study drug administration. 3) The proportion of patients requiring rescue sedation during 24 h of study drug administration. 4) The total cumulative dose of rescue sedative during 24 h of study drug administration. 5) The time to successful weaning and ICU discharge during 24 h of study drug administration. 6) The weaning success rate and the proportion of patients discharged from the ICU.

**Clinical Trial Registration:**

https://clinicaltrials.gov/ct2/show/NCT06785571, identifier NCT06785571.

## Introduction

Effective analgesia and sedation are central to the management of critically ill patients, not only to alleviate pain and distress but also to facilitate tolerance of invasive procedures, optimize ventilatory synchrony, and prevent the harmful physiological consequences of uncontrolled nociception. Current international guidelines consistently endorse an “analgesia-first” or “analgosedation” approach, emphasizing that adequate pain control should form the foundation upon which sedation strategies are built—particularly for managing anxiety and achieving amnesia when necessary (Devlin et al., 2018; Barr et al., 2013; Vincent et al., 2016; Society Of Critical Care Medicine Chines e Medical A, 2018). Within this framework, the selection of an appropriate analgesic agent is critical, requiring careful consideration of efficacy, safety, and predictable pharmacokinetics in the context of the unstable physiology commonly seen in intensive care unit (ICU) patients.

Among available pharmacological options—including opioids, non-opioid central analgesics, nonsteroidal anti-inflammatory drugs, and local anesthetics—nalbuphine hydrochloride exhibits a particularly distinctive pharmacological profile. Developed by DuPont in 1965 and approved for clinical use in the United States in 1979 under the brand name Nubain®, nalbuphine hydrochloride acts as a κ-opioid receptor agonist and μ-opioid receptor antagonist (Schmidt et al., 1985). Its dual mechanism provides effective analgesia while demonstrating a ceiling effect on respiratory depression, along with favorable hemodynamic stability and a low potential for abuse (Schmidt et al., 1985; Mathai et al., 2019). Clinically, nalbuphine hydrochloride has been widely used for the management of moderate to severe pain (Schnabel et al., 2014), as an adjunct in perioperative anesthesia (Kubica-Ciel et al., 2015; Liu et al., 2022), and as a safe and effective analgesic during labor (Vavri et al., 2005). Given these characteristics, nalbuphine hydrochloride represents a theoretically favorable alternative in clinical settings where the limitations of conventional opioids are of significant concern—particularly among critically ill patients.

Indeed, although traditional opioids remain mainstays of analgesic therapy in the ICU, they are associated with notable adverse effects. Dose-dependent respiratory depression poses ongoing risks even in mechanically ventilated patients, suppression of gastrointestinal motility may delay recovery, and growing evidence links their use to long-term dependence and increased cumulative opioid exposure (Devlin et al., 2018; Müller-Lissner et al., 2017; Donohue et al., 2019). These drawbacks highlight the need for viable therapeutic alternatives. Nalbuphine may offer a favorable safety profile in ICU patients due to the above advantages. However, high-quality clinical data supporting its use specifically in ICU patients remain limited. Critical illness frequently alters drug absorption, distribution, metabolism, and excretion (Chen and Huang, 2024), and analgesic requirements in this population may differ significantly from those observed in surgical or obstetric cohorts, thus necessitating targeted evaluation. To address these evidence gaps, we conduct a clinical study to evaluate the efficacy and safety of nalbuphine hydrochloride for analgesia in ICU patients. To rigorously evaluate its role, hydromorphone hydrochloride, a potent μ-opioid agonist widely used in critical care analgesia (Liu and Gropper, 2003), was selected as the positive control to determine whether nalbuphine can provide non-inferior analgesia while reducing the adverse effects commonly associated with traditional opioids. Collectively, our study provides essential evidence for nalbuphine’s potential role in critical care analgesia.

## Methods

### Study design

This is a multicenter, randomized, single-blind, parallel, positive control study initiated by Yichang Humanwell Pharmaceutical Co., Ltd., China. Apart from our ICU, 15 ICUs from tertiary hospitals in China enroll patients in the trial. This study was registered at ClinicalTrials.gov (NCT06785571). The trial protocol is reported in accordance with the Standard Protocol Items: Recommendations for Interventional Trials (SPIRIT) guidelines (Calvert et al., 2018). Table 1 presents the scheduled process of enrollment, interventions, and measurements.

**TABLE 1:** Scheduled process of enrollment, interventions, and measurements.

Timepoint	Study period				
	Enrollment	Allocation	Post-allocation		Close-out
	After admission to ICU	After obtaining informed consent	Before study drug administration	Study drug administration	12–48 h after study drug administration ended
Enrollment					
Eligibility screen	X	​	​	​	​
Informed consent	X	​	​	​	​
Allocation	​	X	​	​	​
Interventions					
Nalbuphine hydrochloride	​	​	​	X	​
Hydromorphone hydrochloride	​	​	​	X	​
Assessments					
Baseline variables	X	​	​	​	​
Vital signs	X	X	X	X	X
RASS score	X	X	X	X	​
CPOT score	X	X	X	X	​
Laboratory tests	X	​	​	​	X
APACHE II score and SOFA score					
Dose of drugs (study drugs, analgesics, vasopressors, rescue sedatives)	​	​	​	X	​
AEs and SAEs	​	​	​	X	​
Outcome variables	​	​	​	​	X

RASS, Richmond Agitation–Sedation Scale; AEs, adverse events; SAEs, serious adverse events.

### Participants

Inclusion criteria are: 1) age ≥18 years and ≤80, 2) ICU patients who are intubated are expected to require mechanical ventilation for more than 6 h, 3) patients or their guardians have a full understanding of the purpose and significance of this trial, and voluntarily participate in this clinical trial and sign an informed consent form.

Exclusion criteria are: 1) allergy or unsuitability to any composition of study drugs or propofol, 2) life expectancy of less than 48 h, 3) neurological disorder and any other condition interfering with sedation assessment, 4) gastrointestinal obstruction, 5) asthmatic, 6) abdominal compartment syndrome, 7) serious hepatic dysfunction (Child-Turcotte-Pugh score 10–15), 8) acute kidney injury (KDIGO stage 2 or 3) or chronic kidney disease with glomerular filtration rate (GFR) < 29 mL/min/1.73 m^2^, 9) circulatory instability (the need for a continuous infusion of norepinephrine at ≥ 0.5 ug/kg/min to maintain proper blood pressure), 10) a need of deep sedation (RASS score −4 or −5) or paralytics, 11) anticipation to receive operations (including tracheotomy), 12) abuse of controlled substances or alcohol, 13) pregnancy, lactation, or an intention of gestation in 6 months, 14) inclusion in another interventional trial in the past 30 days, 15) other conditions deemed unsuitable to be included.

### Randomization and blinding

Eligible patients are allocated at a 1:1 ratio to the experimental or control group, with competitive enrollment across centers. The randomization sequence is generated and assigned via the Interactive Web Response System (IWRS). A single-blind design is used.

### Intervention

Analgesics and sedatives administered prior to enrollment are discontinued. In the experimental group, patients receive intravenous nalbuphine hydrochloride (Yichang Humanwell Pharmaceutical Co., Ltd., China). A loading dose of 6 mg nalbuphine Hydrochloride is administered over 30 s (±10 s), followed immediately by initiation of a continuous maintenance infusion at a baseline rate of 2.0 mg/h. Five minutes after the loading dose, if the patient’s Critical-Care Pain Observation Tool (CPOT) score is greater than 2, a 2 mg supplemental bolus is given. This supplemental bolus may be repeated once, with a minimum interval of 5 min between doses, allowing up to two supplemental boluses within the first 20 min. The total dose, including the loading dose and any supplemental boluses, is capped at 10 mg, and no additional boluses are permitted beyond the initial 20-min period. Twenty minutes after the loading dose or once the cumulative dose reaches 10 mg, the maintenance infusion rate is titrated according to CPOT and Richmond Agitation-Sedation Scale (RASS) scores. Dose adjustments are made in increments of 0.5 mg/h, within a target range of 1.0–5.0 mg/h, with a minimum of 5 min between each adjustment. In the control group, patients receive hydromorphone hydrochloride (Yichang Humanwell Pharmaceutical Co., Ltd., China) via continuous intravenous infusion, initiating at 0.5 mg/h. The infusion rate is adjusted based on CPOT and RASS scores in increments of 0.5 mg/h within a target range of 0.5–3.0 mg/h, with at least 5 min between adjustments.

If a patient’s CPOT score remains above 2 despite reaching a nalbuphine infusion rate of 3.0 mg/h or a hydromorphone hydrochloride infusion rate of 2.0 mg/h, rescue sedation with propofol is initiated. Propofol is started at an initial infusion rate of 0.5 mg/kg/h and subsequently titrated in increments of approximately 25% of the current rate (0.125 mg/kg/h), within a dosage range of 0–4 mg/kg/h.

The study drug infusion is discontinued after 24 h (±10 min) of administration or earlier if the patient met weaning criteria and a spontaneous breathing trial is planned. Patients requiring ongoing analgesia or sedation after discontinuation of study drugs are managed at the investigator’s discretion.

### Electrolyte management

Given the well-established influence of electrolyte imbalances on pain perception, sedation efficacy and stability, and cardiovascular safety (Eisinger et al., 2022), serum concentrations of key electrolytes—including potassium, magnesium, calcium, and phosphate—will be measured at baseline and monitored daily throughout the study duration. Any clinically significant deviations from reference ranges will be promptly addressed in accordance with institutional clinical protocols. Patients exhibiting persistent, protocol-intractable electrolyte abnormalities—defined as those that remain uncorrected despite adherence to standard management guidelines and that may compromise the reliability of sedation assessments or elevate the risk of adverse cardiac or neurological events—will be systematically documented and included in the prespecified safety analyses.

### Outcomes

The primary outcome is the analgesic success rate during 24 h of study drug administration. Analgesic success is defined as the fulfillment of both criteria: 1) no administration of rescue analgesic medication, and 2) maintenance of a CPOT score ≤2 for at least 70% of the observation period during study drug infusion.

The secondary outcomes include: 1) The percentage of time within CPOT score ≤2 and RASS score between −2 and +1 during 24 h of study drug administration: This metric represents the proportion of time during which a patient maintains a CPOT score of ≤2 and a RASS score between −2 and +1, relative to the total duration of study drug administration. 2) The frequency and dosage of rescue sedation during 24 h of study drug administration: The frequency refers to the number of rescue sedative administrations, while the dosage indicates the total amount of propofol administered as rescue sedation during the infusion of the study drug. 3) The proportion of patients requiring rescue sedation during 24 h of study drug administration: This is the percentage of patients in each treatment group who received concomitant propofol for rescue sedation during study drug infusion, calculated as (Number of patients receiving propofol/Total number of patients in the group) × 100%. 4) The total cumulative dose of rescue sedative during 24 h of study drug administration: The sum of all propofol doses administered as rescue sedation during the period of study drug infusion. 5) The time to successful weaning and ICU discharge during 24 h of study drug administration: Time to successful weaning is defined as the interval from the initiation of the study drug to liberation from mechanical ventilation. Time to ICU discharge is defined as the interval from study drug initiation to actual ICU discharge. Successful weaning is achieved when the patient is extubated and subsequently maintains stable spontaneous breathing without the need for reintubation. 6) The weaning success rate and the proportion of patients discharged from the ICU: The weaning success rate reflects the percentage of patients successfully weaned from mechanical ventilation. The proportion of patients discharged from the ICU indicates the percentage of patients transferred out of the ICU within the observation period.

Safety outcomes include adverse drug reactions or adverse events occurring during the study. All patients are monitored throughout the trial period for any adverse events. The clinical manifestations, severity, time of onset, duration, interventions, and outcomes are recorded, and the potential relationship to the study drugs is assessed.

### Data collection and management

Upon enrollment, detailed patient demographic data (age, sex, body weight, ethnicity), medical history, clinical data (vital signs, ECG, laboratory tests), and comorbidities are collected. Vital sign monitoring includes heart rate, systolic and diastolic blood pressure (SBP/DBP), respiratory rate, and peripheral capillary oxygen saturation (SpO2). These parameters are recorded immediately at the initiation of the study drug administration (designated as T0), with subsequent assessments performed every 20 min during the first 2 h post-administration. From 2 to 24 h post-administration, or when weaning screening criteria are met and a weaning trial is planned, recordings are taken every 2 h. Additional recordings are obtained immediately following successful weaning and/or ICU discharge. Body temperature is recorded at T0 and again at 24 h post-administration or when weaning criteria are met. Continuous ECG monitoring is conducted throughout the study drug administration. Standard 12-lead ECG is obtained, and blood samples (for complete blood count, biochemistry, coagulation profile, and pregnancy test when applicable) are collected before randomization and within 12–48 h after discontinuation of the study drugs.

The CPOT and RASS scores are evaluated as baseline assessments prior to randomization. Routine evaluations are performed immediately after T0 and every 2 h thereafter. Prior to any dose adjustment, both scores are documented. After each adjustment, assessments are repeated every 5 min until clinical targets are met and no further dose modifications are needed. If the patient is in undisturbed sleep, the RASS score is recorded as unchanged from the previous value and annotated as “sleeping”. However, for final determination of titration cessation, patients are awakened for formal CPOT and RASS evaluation.

Daily records include cumulative sedative and analgesic doses, time to successful weaning, and ICU discharge time. All adverse events and concomitant medications are systematically recorded throughout drug administration and within 12–48 h after drug discontinuation. A trained investigator at each study center is responsible for the daily screening and enrollment of patients, ensuring strict adherence to the study protocol, and completing the electronic case report form (e-CRF). All data are handled with confidentiality and anonymized to protect patient identity. Access to data is restricted to authorized personnel only. Data accuracy is subject to periodic monitoring and verification by the principal investigator to ensure compliance with regulatory and quality standards.

### Safety evaluation

Safety evaluation encompasses continuous monitoring of vital signs throughout the study period, as well as the systematic observation and documentation of all adverse events (AEs) and serious adverse events (SAEs). AE is defined as any untoward medical occurrence in a patient administered the study drug, which may present as symptoms, signs, illness, or laboratory abnormalities, regardless of a causal relationship with the study drug. SAE is defined as any untoward medical occurrence that occurs after administration of the study drug and results in one or more of the following outcomes: death, life-threatening condition, persistent or significant disability or incapacity, hospitalization or prolongation of existing hospitalization, or congenital anomaly or birth defect. Particular attention will be directed toward electrolyte-related adverse events, including QT interval prolongation, cardiac arrhythmias, and neuromuscular manifestations such as hyperreflexia, muscle cramps, or tetany, which will be systematically recorded and analyzed. All adverse events are to be managed promptly upon detection. Any serious adverse event must be reported to the clinical trial site and the institutional ethics committee within 24 h of awareness.

### Estimation of sample size

The sample size estimation is based on a non-inferiority design. According to previous findings and literature reports (Liu et al., 2018), nalbuphine is capable of maintaining the target analgesic effect within 3–24 h post-administration, with CPOT scores ≤2 for more than 70% of the time. An analgesic success rate approaching 100% is anticipated. We estimate an efficacy of 99% in the experimental group, and the control groups. Following consultation of several experts, the non-inferiority margin δ is set at −8%, meaning that an 8% reduction in efficacy representing the maximum clinically acceptable difference. A two-sided significance level of α = 0.05 and a power of 80% (β = 0.2) are applied. Sample size estimation is conducted using the Farrington and Manning method via PASS 2022 software, assuming a 1:1 allocation ratio between the two groups, and 73 participants per group is required. Assuming an anticipated dropout rate of approximately 12%, a total of 168 subjects is needed.

### Statistical analysis

Statistical analyses will be performed using SAS software (version 9.4 or higher). Continuous variables will be presented as mean ± standard deviation for normally distributed data and median ± interquartile range for non-normally distributed data, whereas categorical variables will be summarized as frequencies and percentages. All statistical tests and confidence intervals (CI) will be two-sided, with statistical significance defined as a p-value less than 0.05. The difference in analgesic success rates between the experimental and control groups will be estimated using the Miettinen and Nurminen method to calculate the two-sided 95% CI. Non-inferiority will be established if the lower bound of this interval exceeds −8%. The proportion of time during which patients maintain a CPOT score ≤2 and a RASS score between −2 and +1 will be analyzed using the arcsine square root transformation, followed by between-group comparison via the t-test. Time from initiation of study drug to successful weaning and time to ICU discharge will be illustrated using Kaplan-Meier survival curves and compared using the log-rank test. The frequency and dosage of rescue propofol sedation, along with total propofol dose administered, will be compared between groups using either the t-test or Wilcoxon rank-sum test, depending on data distribution. The proportions of patients requiring concomitant propofol sedation, weaning success rate, and ICU discharge rate will be compared using the χ^2^ test or Fisher’s exact test. The incidence of adverse events will also be assessed using χ^2^ test or Fisher’s exact test.

### Ethics approval and informed consent

The study protocol was approved by the Ethics Committee of Wuhan Union Hospital (approval number: 0407-2023) and will be approved by all participating centers. All procedures will be conducted in accordance with the principles of the Declaration of Helsinki. Written informed consent will be obtained from all patients or their legally authorized representatives prior to enrollment. Participation is entirely voluntary, and patient confidentiality will be strictly maintained throughout the study. Patients or their legally authorized representatives retain the right to withdraw from the study at any time and for any reason, without any impact on their subsequent medical care.

### Dissemination

Research data may only be disclosed or presented with the prior approval of the principal investigator. Study findings will be published in peer-reviewed journals and disseminated to the public.

## Discussion

This protocol outlines a phase III, randomized, single-blind, parallel, positive-controlled clinical trial designed to evaluate the effectiveness and safety of nalbuphine hydrochloride for analgesia in mechanically ventilated ICU patients. The study is particularly meaningful as it reflects a growing emphasis on individualized and safer analgesia strategies in critical care. If nalbuphine hydrochloride demonstrates analgesic efficacy comparable to hydromorphone hydrochloride while offering a more favorable safety profile, it can provide clinicians with an important alternative to ICU analgesia. Such results could help reduce reliance on deep sedation and limit risks associated with prolonged exposure to full μ-agonist opioids. While the design of this study incorporates several notable strengths, it also has inherent limitations.

### Strengths

A major strength of this trial is its robust and pragmatic phase III design. This design ensures that the study is adequately powered to provide conclusive, real-world evidence on the efficacy and safety of nalbuphine hydrochloride in ICU patients. The randomized, parallel-group, positive-controlled methodology strongly enhances internal validity. Randomization minimizes selection bias and ensures comparable baseline characteristics between groups, thereby strengthening the causal inference between the intervention and the outcomes. The use of hydromorphone hydrochloride—rather than a placebo—is both ethically appropriate and clinically relevant for managing pain in critically ill patients. It enables direct comparison of nalbuphine’s performance against a current standard of care, which is the most meaningful question for clinicians. Another strength lies in the selection of the patient population and endpoints. ICU patients frequently experience complex and undertreated pain, representing a significant unmet clinical need (Puntillo et al., 2014). Use of validated tools such as the CPOT (Gélinas et al., 2006) ensures standardized and reliable pain assessment. Finally, nalbuphine’s low abuse potential has broader implications for public health. Its expanded use in ICU settings may support global efforts to reduce opioid misuse and dependence.

Beyond these methodological strengths, nalbuphine’s distinct pharmacological profile—as a selective κ-opioid receptor agonist and partial μ-opioid receptor antagonist—confers intrinsic opioid-sparing properties that are consistent with contemporary clinical imperatives to reduce cumulative opioid exposure in critically ill patients (Maciel et al., 2023). This mechanism may attenuate the incidence and severity of adverse effects associated with full μ-opioid agonists, including postoperative ileus, dose-dependent respiratory depression, and the development of analgesic tolerance. Furthermore, because the intervention protocol defines pain and sedation targets using validated, objective assessment instruments, it may be applicable across diverse ICU populations, including medical, surgical, and trauma patients. In specialized units—such as neurosurgical and cardiothoracic ICUs—nalbuphine’s favorable hemodynamic neutrality and minimal impact on level of consciousness may confer additional therapeutic advantages. Nevertheless, prespecified stratified analyses by primary admission diagnosis and ICU subspecialty will be critical to rigorously evaluate generalizability and inform context-specific implementation.

### Limitations

Despite these strengths, several limitations merit consideration. First, although nalbuphine exhibits a ceiling effect for analgesia, this occurs at doses exceeding 0.4 mg/kg, which are not reached in our protocol (maximum 10 mg loading plus 5 mg/h maintenance). Within the selected dose range, dose-dependent analgesic efficacy is preserved. Nevertheless, the ceiling effect may limit efficacy in patients with very high opioid requirements, which we have acknowledged as a study limitation. Second, one limitation of our study is the lack of precise equipotent dosing between nalbuphine and hydromorphone. The chosen dose ratio (approximately 4:1) may underestimate nalbuphine’s required dose if the true potency ratio is 5-7.5:1. To mitigate this, our protocol permits supplemental boluses and upward titration to a maximum of 5 mg/h. Moreover, we will conduct sensitivity analyses using alternative potency assumptions to assess the robustness of the non-inferiority conclusion. Future dose-optimization studies in ICU patients are warranted. Third, the protocol, as described, may face challenges related to generalizability. ICUs are highly heterogeneous (Zimmerman et al., 2006), and patient populations can vary significantly between centers in terms of primary diagnoses (e.g., medical, surgical, or trauma), severity of illness, and baseline organ function. If the inclusion and exclusion criteria are too restrictive, the results may not be applicable to all subtypes of critically ill patients. Additionally, the findings will be directly comparable only to the specific positive-control analgesic chosen, conclusions may not extend to all opioids or analgesics used in the ICU. The single-blind design carries an inherent risk of assessment bias despite mitigation strategies, and the exclusion of patients with severe organ dysfunction, hemodynamic instability, or need for deep sedation further limits generalizability to the sickest ICU subpopulations. Variability in patient pharmacodynamics—especially in those with organ dysfunction, comorbidities, or concurrent sedative use—may also influence analgesic response. Moreover, the 24-h intervention period may not capture longer-term outcomes such as delirium, opioid withdrawal, or post-ICU dependence, and variations in institutional sedation protocols and nursing expertise could influence implementation fidelity across centers. These factors should be considered when interpreting results and may inform future research aimed at optimizing dosing strategies or evaluating specific subgroups.

To maximize clinical utility, prespecified subgroup and sensitivity analyses are necessary. Subgroup analyses stratified by baseline hepatic function (e.g., Child-Pugh class) and renal function (e.g., KDIGO stage) would assess whether nalbuphine’s noninferiority is maintained across the spectrum of organ dysfunction severity. Sensitivity analyses adjusting for key potential confounders—including illness severity (e.g., APACHE II score), concomitant sedative use, and delirium status—would further enhance the robustness and generalizability of the primary findings. Although these analyses are exploratory, they are valuable for generating new research ideas and guiding personalized pain management in critically ill patients.

## Conclusion

In summary, this Phase III clinical trial represents a critical step toward establishing evidence-based use of nalbuphine for ICU analgesia. By rigorously comparing nalbuphine with a standard opioid analgesic, the study aims to clarify its potential advantages, define its safety profile, and contribute to evolving paradigms of patient-centered and safer analgesia in critical care. The findings have the potential to expand therapeutic options, improve pain management practices, and enhance outcomes for a vulnerable and complex patient population.

## Data Availability

The raw data supporting the conclusions of this article will be made available by the authors, without undue reservation.
